# Cytomegalovirus in Indian systemic lupus erythematosus patients: troublemaker or onlooker?

**DOI:** 10.11604/pamj.2020.37.38.18836

**Published:** 2020-09-09

**Authors:** Shalini Dubey, Camilla Rodrigues, Chaitali Nikam, Rohini Samant

**Affiliations:** 1 Research Laboratories, P.D. Hinduja Hospital and Medical Research Centre, Mumbai, India,; 2 Rheumatology Department, P. D. Hinduja Hospital and Medical Research Centre, Mumbai, India

**Keywords:** Systemic lupus erythematosus, cytomegalovirus, real-time polymerase chain reaction, reactivation

## Abstract

**Introduction:**

cytomegalovirus (CMV) infection has been reported to be associated with onset/exacerbation of systemic lupus erythematosus (SLE). In an attempt to verify this, we studied CMV infection in SLE patients.

**Methods:**

forty-two SLE patients were studied at 3-time points; disease onset/flare, at peak of immunosuppression (at 6 weeks) and at low doses of immunosuppression (at 6 months). We studied healthy blood donors as controls, only once. Clinical assessment and SLE Disease Activity Index scoring were done at each visit. RT-PCR and ELISA were performed to detect CMV viral-load and anti-CMV antibodies (Ab) respectively.

**Results:**

nine of 106 patients had detectable viral-load (145-50,000 copies/ml). Of these nine, three patients had significant viral-load, 6 patients had low viral-loads of doubtful clinical significance. None of the patients developed CMV disease. Six of 42 cases were positive for IgM Abs. All controls were negative for CMV DNA as well as CMV IgM Abs. All samples from patients and controls were positive for CMV IgG Ab indicating widespread prevalence.

**Conclusion:**

significantly, a higher seroprevalence of CMV IgM Abs against CMV observed in SLE patients when compared to controls, indicating possible reactivation due to immune modulation.

## Introduction

Systemic Lupus Erythematosus (SLE or Lupus), is a clinically heterogeneous disease which is autoimmune in origin. It is characterized by the presence of antibodies against nuclear antigens [1,2]. It can affect any organ of the body and has a protean range of manifestations. Several environmental factors such as viruses along with genetic factors have implicated as triggering factors for onset or flare of this disease. The disease also has natural difficulties. Treatment with immunosuppressive drug increases the risk of infections (either bacterial or viral) in patients with SLE. Viral infections can cause more severe complications in an immunosuppressed patient. Persistent viral antigens after an infection may cross-react with antibodies and trigger new onset or flare of SLE [3,4]. Owing to the fact that herpes viruses have the potential of evading host clearance system and remain latent in the host, it becomes essential to study them in individuals with complicated immune disorders like SLE [5-7]. CMV belongs to the herpesviridae family and inherits the ability to evade the host's immune system. Thus, post infection, it remains latent in the host. Since decades, the role of CMV in SLE patients has remained speculate. There are several case reports and a few group studies which point to a role of CMV infection in SLE patients [8-11]. CMV infection: evidence of CMV replication regardless of symptoms. CMV disease: evidence of CMV infection with attributable symptoms. To the best of our knowledge, there are very few longitudinal studies to assess this aspect [12]. Since, CMV is a treatable virus, and evidence of its role in SLE patients might help in taking therapeutic decisions. The present study attempts to determine the relationship of CMV with SLE during new onset, active and stable disease.

## Methods

**Patient selection and demographic details:** SLE patients with flare or new onset of the disease were enrolled in the study. New onset of SLE was diagnosed according to the American College of Rheumatology (ACR) classification criteria. Patients fulfilling minimum four out of 11 criteria were said to have SLE. Total 42 SLE patients and 42 healthy blood donors enrolled as cases and controls respectively. All of these patients satisfy the SLICC-2015 criteria for diagnosis of SLE. In all patients, clinical assessment and SLE Disease Activity Index (SLEDAI) recorded at each visit. Based on the SLEDAI score the patients classified as having a mild, moderate or severe disease. The flare of the disease was defined as an increase in SLEDAI score i.e. >3 when compared to previous SLEDAI score. The blood donors who fulfilled the requisite criteria were enrolled as controls. The controls were not matched for age or gender.

**Sample collection in cases and controls:** blood samples were collected in EDTA vials from all subjects after obtaining informed consent. The plasma was separated from blood by centrifuging the sample at 5000 rpm for 5 minutes. The plasma samples were stored at -80°C until further use. Sample collection from SLE patients was performed at three different time points. The first sample was collected at the time of flare (n = 27) or new onset (n = 15) of disease. The second sample was collected after 6 weeks of the first collection (maximum doses of immunosuppressant i.e. up to 40 mg). The third sample was collected after 6 months of 1^st^ collection (at stable and low doses of immunosuppression). One sample was collected from each control. Treatment details of SLE patients: This cohort of SLE patients was treated with Prednisolone in combination with Mycophenolate mofetile (MMF) and Azathioprine. At the time of new onset or flare of the disease, the patients were given Prednisolone (30-40mg/day), MMF (up to 2gm/day) or Azathioprine (upto150mg/day) for 6 weeks. The dose of Prednisolone was tapered to <10mg/day by the end of 3 months and the patients were asked to continue the steroid sparer drugs. Patients with aggressive renal/neurologic diseases were treated with cyclophosphamide.

**RT-PCR and ELISA Testing:** DNA was extracted from 500µl plasma using Qiamp DNA Blood Mini Kit by Qiagen using manufacturer's protocol. Real-time polymerase chain reaction was performed according to manufacturer's protocol using CMV R Gene kit by Biomereux on ABI 7500 FAST Real-Time Dx System. ELISA assays for IgM and IgG antibody detection were performed using kits provided by Diapro all manufacturers´ instructions were followed. The optimal cut-off value for viral load was approximately 2x10^4^ copies/ml.

**Statistics:** Spearson´s correlation, student's t-test and Chi square tests were intended to check the statistical significance of CMV viral load and anti-CMV antibodies respectively, between SLE patients and controls.

## Results

**Figure 1:**
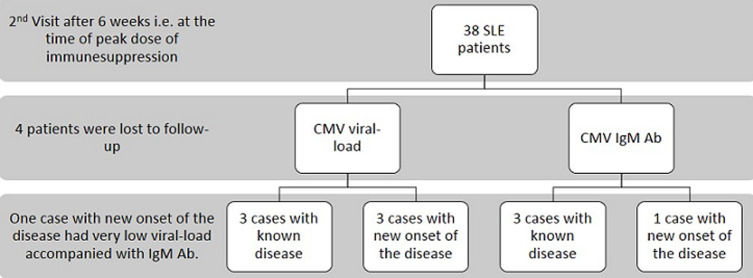
results for first visit

**Demographic details of SLE patients:** in SLE patients, 40 out of 42 patients were females and 2 were males. Both the male patients were known cases of SLE, whereas, of 40 female patients, 15 had new onset of the disease and 25 were known cases of SLE. Fifteen cases were known to be negative for HIV infection, whereas remaining 27 were not tested for the same. One known case had documented a history of CMV related illness. Eight patients for 2^nd^ visit and 4 patients for 3^rd^ visit were lost to follow up (Figure 1). We classified the patients based on organ involvement. At the time of enrolment, more than 50% patients had complaints of fever, malar rash and arthritis. More than 30% patients had anaemia, alopecia, mucosal ulcers, proteinuria, and leukopenia. Myositis and vasculitis were rare complaints (Table 1). In current study, we calculated and compared the SLEDAI score for each patient at each visit. The SLEDAI of the patients were observed to be on higher side at the first visit i.e. at flare/new onset of the disease but it gradually reduced in most of patients, after the treatment at second and third visits. We did not find any correlation between SLEDAI score and anti-CMV IgG antibodies (*r^2^=0.007, p=0.960*). In this study, we enrolled twenty-seven known SLE patients with flare and 15 patients with new onset of the disease. Table 2 shows the percentage of patients positive for CMV viral load and anti-CMV IgM Abs, whereas Table 3 describes the clinical details of these patients (Table 2, Table 3).

**Table 1:** demographic details of SLE patients and controls

Characteristics	Cases	Controls
Age at enrolment	32 [10.7]	35 [11.2]
Ratio of M: F weight at enrolment	2: 40	38: 4
New onset n [%]	27 [64.3]	-
Known n [%]	15 [35.4]	
Skin/MAS/Joints n [%]	128 [42.8]	-
Nephritis n [%]	12 [28.5]	-
Neurological involvement	5 [11.9]	-
Hematological manifestations	5 [11.9]	-

**Table 2:** number of patients with positive viral load and IgM Abs

Visit	Total Samples	No of cases with positive CMV viral load; n [%]	No of cases with positive anti-CMV IgM Abs; n [%]
1^st^ At active Disease/Onset	42	2 [4.76]	6 [14.28]
2^nd^ At 6 weeks	34	6 [17.14]	4 [11.76]
3^rd^ At 6 months	30	1 [3.33]	4 [13.33]
Total	106	9	14

**Table 3:** clinical details of patients positive for CMV viral load and anti-CMV IgM Abs

Patient ID	Visit	Viral Load	IgM	IgG	SLEDAI at first visit	Clinical symptoms
		[copies/ml]	Abs	Abs		
P1	2^nd^	50600	-	+	5	Few healing ulcers, rash, joint pain & ankle edema
P2	1^st^ Flare	1445	-	+	5	Oral ulcers, mild sub plural fibrosis, Documented MAS, Recurrence of MAS
P3	1^st^ Onset	1218	-	+	15	Brownish rash all over the body, Lymphadenopathy, hepatomegaly, fatty infiltration, horse shoe shaped kidney, enlargement of liver & spleen, pancytopenia MAS
P4	2^nd^	667	-	+	1	Cough with expectoration
**5/9 patients had <555 copies/ml therefore of doubtful significance**						
P5	1^st^, 2^nd^, 3^rd^	Not Significant	+	+	15	MAS
P10	1^st^	-	+	+	13	Mouth ulcers, breathlessness, swelling & nodules over legs
P11	1^st^	-	+	+	5	Oral ulcers, wrist synovitis & oral candida
P12	1^st^, 2^nd^, 3^rd^	-	+	+	14	Body swelling, low complement levels, proteinuria, hematuria & pyuria
P13	1^st^, 2^nd^, 3^rd^	-	+	+	8	Joint pain & weakness
P14	1^st^, 2^nd^, 3^rd^	-	+	+	15	Mild fever, joint pain, body ache, myalgia, ankle & shoulder pain

**Figure 2:**
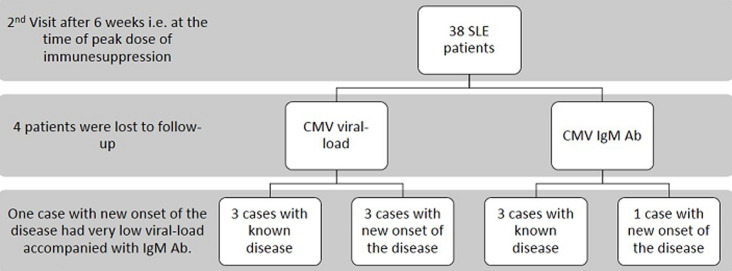
results for second visit

Only two known female cases out of 42 had a detectable viral load of 1218 copies/ml and 1445 copies/ml. Both of them showed features of macrophage activation syndrome (MAS), which responded to immune suppressive treatment. It can only be speculated that CMV was responsible for MAS. None of them had anti-CMV IgM Abs. One new case and four known cases were positive for anti-CMV IgM Abs. All these patients were negative for CMV viral-load. No difference was found in the SLEDAI score of the patient who had a detectable viral load or anti-CMV IgM antibodies positive when compared with CMV negative patients. Three new and three known SLE cases had detectable viral-load of CMV during the 2^nd^ visit. Of these six patients, only one known patient had viral-load of 50,600 copies/ml. Five patients had very low CMV viral-load (>1000 copies/ml). One of the new onset patients with CMV viral-load of 125 copies/ml was also positive of anti-CMV IgM Abs. Three more known cases with undetectable viral-load were also positive for anti-CMV IgM Abs (Figure 2). None of these patients showed any clinical symptoms of CMV infection as diarrhoea, digestive tract ulcers, visual impairment or pneumonia. Only 1 patient with disease onset had detectable CMV viral-load on 3^rd^ visit. None of the patients were positive for anti-CMV IgM Abs during this visit. None of the controls had detectable CMV viral-load or positive anti-CMV IgM Abs. SLE patients [5.19±1.89] had a higher mean value of anti-CMV IgG Ab titre when compared to the controls [2.00±0.19]. [*p=<0.001*] No significant difference was noticed in the CMV viral load and CMV IgG and IgM Abs concentration, amongst patients with different organ involvement.

**Statistical analysis:** Spearman´s correlation was used for determining the correlation of SLEDAI with anti-CMV IgG Abs. Chi Square Test was performed to compare the prevalence of anti-CMV IgG antibodies in SLE patients and healthy blood donors. There was no difference in the prevalence of anti-CMV IgG antibodies between case and control groups. 40/42 SLE patients as well as healthy blood donors were positive for anti-CMV IgG antibody [*p=1*]. Chi Square Test was performed to compare the prevalence of anti-CMV IgM antibodies between case and control group. The difference found was statistically significant [*p=0.011*]. Six out of 42 cases were positive for anti-CMV IgM antibodies at 1^st^ visit, however, none of the controls were found to be positive for anti-CMV IgM antibodies. Student’s-t test was implemented to compare the mean concentration of anti-CMV IgG Abs in cases and controls [*p=<0.001*]. Statistical significance of CMV viral load could not be tested between cases and controls as a maximum number of cases and all controls were negative for this test.

## Discussion

It's been reported, to cause diarrhoea, digestive tract ulcers, retinitis, pneumonia and hepatitis in patients with rheumatic diseases [13,14]. This virus is known to cause significant morbidity and mortality in immunosuppressed individuals like transplant and HIV patients [15]. CMV can cause both systemic and organ specific disease, not only through the direct cytopathic effect of viral replication in a host cell but also through inflammatory processes [16]. CMV causes immunosuppression in its host as it inhibits natural killer (NK) cell activity, adenomatous polyposis coli (APC) differentiation and T cell proliferation in the process of evading the immune system [17]. CMV is also known to initiate autoimmunity either by viral mimicry or non-specific B cell proliferation. Inflammation, stress and lowered immunity are known factors for reactivation of CMV, which may precede CMV disease in susceptible individuals. All these factors are also present in SLE patients and the treatment given to these patients are immunosuppressive, which makes these patients highly susceptible to viral infections and reactivation. Since CMV is a treatable virus, evidence of its role in SLE patients might help in taking therapeutic decisions; hence, we decided to study role of CMV in SLE patients [16]. In the current study, we collected samples at following three-time points: First: at flare or onset of the disease. Second: at peak of immune suppression (after 6 weeks). Third: at low doses of immune suppression (after 6 months). We calculated and compared the SLEDAI score for each patient at each visit. We did not find any correlation between SLEDAI and anti-CMV IgM/IgG antibodies. The SLEDAI of all patients was observed to be high at the first visit i.e. at flare/new onset of the disease but it gradually decreased after the treatment at 2^nd^ and 3^rd^ visits as was expected.

In a study by Bendiksen and colleagues, the disease activity as determined by SLEDAI was monitored approximately monthly for each patient throughout the one year observation period. They did not find any correlation between the SLEDAI and CMV viruria. SLEDAI score did not correlate with CMV IgG or IgM antibody levels in any of the patients [12]. In various case reports, the clinical manifestation of CMV in SLE patient was described as the presence of fever, cough or dyspnoea, abdominal pain or mucous and/or bloody stools and visual disturbance. In the current study patients with detectable viral loads responded to medications prescribed for SLE. None of these patients showed typical manifestations of CMV disease. Nine samples were found to have a detectable viral load of which 6 patients had low viral loads (1000 copies/ml) of doubtful significance. Two out of remaining three had viral loads at the first visit and both of them showed features of macrophage activation syndrome (MAS), which responded to immune suppressive treatment. It can only be speculated that CMV was responsible for MAS. One male patient had a viral load of 50,600 copies/ml at second visit but was completely asymptomatic. The patients who tested positive on viral load or serological testing did not show any clinical features of CMV related illness or CMV disease. The phenomenon of patients being asymptomatic in spite of having a high viral load in plasma is not new. Many transplant studies have also reported this phenomenon. In current study, one of our patients showed high viral load but was asymptomatic [18].

It has been reported that CMV might be present at the local site of infection despite low viral load in the plasma. In our study, six patients had very low levels of viral loads which were clinically not significant. However, these patients also did not have manifestations suggestive of focal CMV disease [16]. In the current study, we intended to see CMV viral load and anti CMV antibodies as a direct evidence of CMV reactivation or new infection in all SLE patients. We did not study focal CMV disease. Six patients tested positive for IgM antibody against CMV whereas all the controls were found to be negative for it. The prevalence of IgG antibodies was found to be 95% in both the groups [19,20]. Of the 6 patients who tested positive for anti-CMV IgM antibodies at 1^st^ visit, 3 patients had joints involvement. From above mentioned 3 cases, 2 patients also had rashes and mucosal ulcers, 1 patient had renal involvement. The third patient who was positive for anti-CMV IgM antibody also had a detectable low viral load (125 copies/ml) at second visit. At the time of presentation, this patient had shown reactivation of MAS. The presence of CMV IgM antibody indicates acute infection and IgG antibody indicates chronic infection but the presence of both the antibodies simultaneously indicates a recent infection or reactivation of CMV [21]. Amel and colleagues reported a case study of 22-year-old women with newly diagnosed with SLE due to CMV induced central nervous system (CNS) vasculitis; implicating CMV as a potential trigger for SLE [22]. More than 90% of our cases and controls were found to be positive for CMV IgG antibody, which indicates that CMV is a very common virus in Indian population. However, in our study population, we found that prevalence of CMV IgM antibody was higher in the case group when compared to control group. All these IgM positive cases were also positive for IgG antibodies; this, however, shows that the rate of CMV reactivation was more in SLE patients when compared to healthy controls [23].

## Conclusion

A causal role for CMV in SLE patients could not be established in our study but based on the literature review and high IgM positivity in these patients, the role of CMV in SLE onset or its exacerbation cannot be ruled out. It has been reported that SLE patients can show false positive IgM antibodies against CMV. Hence, the role of CMV in SLE as a troublemaker or onlooker; remains unanswered and requires further studies.

### What is known about this topic

- Several studies have implicated as a triggering factor for SLE flare and new onset of the disease;
- CMV is known to be actively involved in the inflammatory responses in the autoimmune diseases and vice versa.

### What this study adds

- To the best of our knowledge, current longitudinal study is first of its kind, looking at the aspect of role of CMV in SLE patients at the highest and lowest doses of immunesuppressives;
- Present study also brings it to the notice that slight shedding of the virus and reactivation is often observed in SLE cases due to CMV's participation in inflammation induced autoimmune reactions.
